# Predictive value of geriatric-quickSOFA in hospitalized older people with sepsis

**DOI:** 10.1186/s12877-021-02182-1

**Published:** 2021-04-13

**Authors:** Francesca Remelli, Federico Castellucci, Aurora Vitali, Irene Mattioli, Amedeo Zurlo, Savino Spadaro, Stefano Volpato

**Affiliations:** 1 Department of Medical Sciences, University of Ferrara, Ferrara, Italy; 2 Geriatrics Unit, Azienda Ospedaliero- universitaria di Ferrara, Ferrara, Italy; 3 Anestesiology and Resuscitation Unit, Department of Morfology, Surgery and Sperimental Medicine, University of Ferrara, Ferrara, Italy; 4 Orthogeriatrics Unit, Azienda Ospedaliero-Universitaria di Ferrara, Via Aldo Moro, 8, 44124 Ferrara, Italy

**Keywords:** Elderly, Sepsis, Delirium, QuickSOFA, Geriatric-quickSOFA

## Abstract

**Background:**

QuickSOFA, a prognostic score proposed for patients with infection, has shown a poor predictive value in the geriatric population, probably because of the inappropriateness of the Glasgow Coma Scale (GCS) in assessing acute alteration of mental status in older patients. Indeed, the GCS might result chronically low in older patient with pre-existing cognitive disorders. The aim of this study was to develop an alternative quickSOFA (geriatric-quickSOFA), using the presence of delirium, assessed according to DSM-5 criteria, instead of GCS assessment, to predict mortality in hospitalized older patients with sepsis.

**Methods:**

Retrospective observational study in Acute Geriatrics Unit of St. Anna Hospital of Ferrara (Italy). The study enrolled 165 patients hospitalized between 2017 and 2018 with diagnosis of sepsis or septic shock. Demographic, clinical data and 30-day survival were collected for each patient. Based on arterial blood pressure, respiratory rate, and the presence of delirium, geriatric-quickSOFA was calculated at admission. Primary outcome was 30-day mortality.

**Results:**

One hundred sixty-five patients were enrolled with a median age of 88 years; 60.6% were men. High quickSOFA score was not significantly correlated neither with in-hospital nor 30-day mortality. High geriatric-qSOFA score was significantly related to both in-hospital (13.3%vs 51.5%, *p* = 0.0003) and 30-day mortality (30.0%vs 84.3%, *p* < 0.00001).

**Conclusion:**

Geriatric-quickSOFA is significantly associate with short-term mortality risk in older patients with sepsis. Geriatric quickSOFA seems to represent a more suitable and useful predictive tool than the traditional quickSOFA in the geriatric population.

## Background

Sepsis is a major public health problem in older people being one of the most common cause of hospitalization, disability and death [1–4]. A recent retrospective population-based study has shown that death due to sepsis is increasing over time, explaining more the one third of all deaths among patients aged 85 and older [5]. Early identification of high risk patients is essential for better prognosis and survival, but in older patients both the diagnosis and prognosis of sepsis are challenging, because of the high variability of clinical presentations with atypical symptoms including, confusional status and delirium [6–10].

For an early recognition of high risk patients with sepsis, a simplified version of the original SOFA, quickSOFA (qSOFA), score has been proposed [11, 12] based on the presence of arterial hypotension, tachypnea and altered mental status defined as a Glasgow Coma Scale (GCS) ≤14.

Few studies have investigated the predictive value of the qSOFA in older patients, mainly in Intensive Care Unit (ICU) wards, [13–19] providing discordant results and suggesting only a weak correlation between qSOFA score and mortality risk. One explanation for the poor predictive value might be the inability of the GCS in assessing mental status changes and fluctuations of acutely ill patients when affected by pre-existing cognitive decline and/or delirium. For example, a patient with severe cognitive impairment might be categorized as having a low GCS because unable to answer simple verbal questions, regardless of the acute effect of sepsis on mental status.

The aim of our study was, therefore, to develop a modified version of the qSOFA to be used in older patient. We hypothesized that a score including the presence of delirium instead of abnormal GCS might have better prognostic value because more specific in assessing the acute mental impairment often present in older patients with sepsis.

## Methods

### Participant recruitment

This is a retrospective study carried out in the Acute Geriatrics Unit of the University Hospital of Ferrara (Italy) between January 2017 and December 2018. The study enrolled patients discharged with diagnosis of sepsis or septic shock. The data for patient selection were obtained from hospital database system. Diagnosis of sepsis was verified according to *sepsis-3* criteria [1]. From the initial sample of 243 patients, 183 clinical records were available via hospital database.

Demographic characteristics and pre-admission functional status were collected from clinical interview. Cognitive status was assessed at admission using the Short Portable Mental Status Questionnaire (SPMSQ) and GCS; the diagnosis of delirium was performed according to Diagnostic and Statistical Manual of Mental Disorders 5th (DSM-5th) Edition criteria [20]. Among chronic diseases, congestive heart failure, diabetes, cancer and cognitive decline were collected; comorbidity was assessed using the Charlson Comorbidity Index (CCI) [21]. All drugs currently taken by the patients on admission were recorded; number of drugs taken was also calculated.

Information regarding clinical chemistry tests were collected; white blood cells, hemoglobin, platelet count, creatinine, bilirubin, albumin, pH, C-reactive protein, procalcitonin were considered in the analyses.

### Prognostic scores and outcomes

**Fig. 1:**
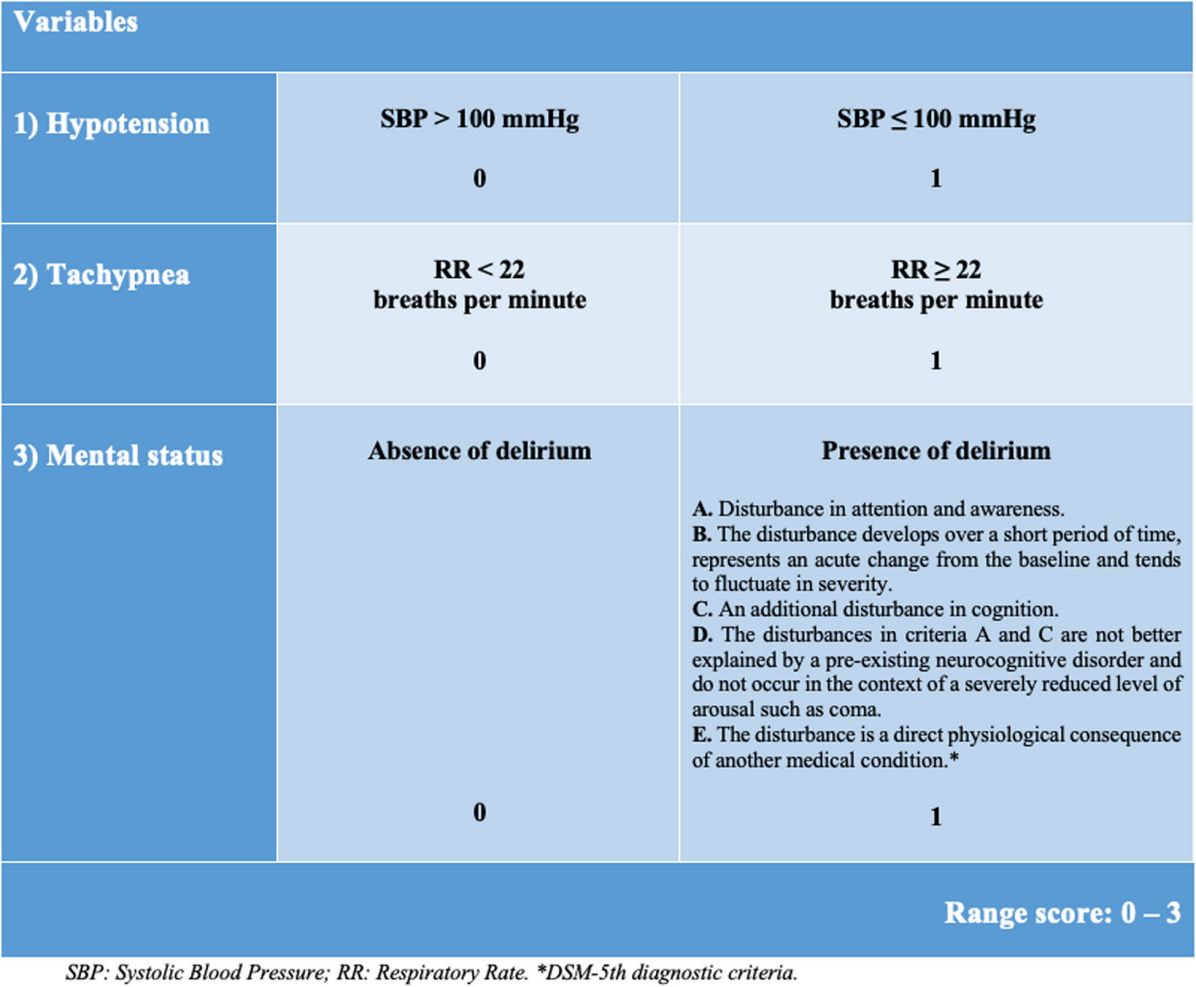
Geriatric-quick SOFA

For each patient, arterial blood pressure, respiratory rate, mental status, evaluated through both GCS and presence of delirium were collected. Using this information, traditional quickSOFA and geriatric-quickSOFA were calculated. Geriatric-quickSOFA was based on the evaluation of three clinical parameters: arterial hypotension (Systolic Arterial Pressure ≤ 100 mmHg), tachypnea (Respiratory Rate ≥ 22/min) and presence of delirium assessed by DSM-5th criteria. To each of these items the value “1” or “0” was assigned whether the sign was present or absent, respectively (score range 0–3), Fig. 1. Because of missing data, qSOFA calculation was possible in 165 patients that represent the final study sample.

The primary outcome of the study was 30-day mortality, collected using hospital database; secondary outcome was in-hospital mortality.

### Statistical analysis

The main demographic and clinical features of patients enrolled were presented using mean and standard deviation for continuous variables; frequency and percentage for dichotomous ones. The characteristics of patients were compared according to in-hospital and 30-day mortality, using Student’s t-test and Pearson’s Chi-Square test for continuous and categorical variables, respectively.

The predictive performance of variables related to the primary outcome was assessed using multivariable logistic regression analysis and expressed through Odds Ratio with 95% Confidence Interval (OR, 95% CI). The better cut-off of geriatric-qSOFA score was evaluated through estimation of sensibility, specificity, prognostic OR, accuracy, and Youden index for both in-hospital and 30-day mortality. *P*-value < 0.05 were considered statistically significant.

Statistical analysis was performed using Software R.

## Results

Median age of the 165 patients enrolled in the study was 88 years; 60.6% (*n* = 100) were men. Most of patients enrolled had cognitive decline (71.5%) with different stages of severity and impaired pre-admission functional status (median of Basic Activities of Daily Living and Instrumental Activities of Daily Living preserved were 1/6 and 1/8, respectively); 53.3% of the sample was bedridden before hospital admission; delirium occurred in over 60% of patients. All patients were affected by pre-existing multimorbidities with a median CCI of 4 out of 32 points. The most frequent primary sites of infection causing sepsis were urinary tract (38%) and lungs (34%). Thirty-seven percent of the sample (*n* = 61) had a qSOFA≥2. All-cause in-hospital mortality was 44.3% (*n* = 73) and 30-day mortality was 75.2% (*n* = 124).

The clinical and demographic characteristics of the population according to 30-day mortality are summarized in Table 1. Clinical characteristics significantly correlated with 30-day mortality were bedridden status (34.2% vs 58.9%, *p* = 0.009), presence of tachypnea (17.1% vs 35.5%, *p* = 0.04), delirium (41.5% vs 68.6%, *p* = 0.002), and low hemoglobin and albumin serum levels (12.1 vs 11.1 g/dl, *p* = 0.02 and 2.8 vs 2.4 g/dl, *p* = 0.004, respectively).

**Table 1:** Selected baseline characteristics of the population according with 30-day mortality

Variables	Survivors (*n* = 41)	Deaths (*n* = 124)	*p*
Age, mean ± *SD*	87.2 ± 4.5	87.6 ± 5.7	0.668
Male sex, *n (%)*	29 (70.7)	71 (57.3)	0.196
Charlson Comorbidity Index, median [IQR]	5 [3, 6]	4 [3, 5]	0.433
Cancer, *n (%)*	6 (14.6)	23 (18.6)	0.723
Diabetes, *n (%)*	17 (41.5)	28 (22.5)	0.129
Chronic heart failure, *n (%)*	15 (36.6)	48 (38.7)	0.926
Cognitive decline, *n (%)*	26 (63.4)	91 (73.4)	0.273
Bedridden, *n (%)*	14 (34.2)	73 (58.9)	0.009
SPMSQ, median [IQR]	9 [5, 10]	10 [6, 10]	0.219
Number of drugs, median [IQR]	6 [5, 9]	7 [5, 9]	0.897
BADL, median [IQR]	1 [0, 1]	0 [0, 1]	0.614
IADL, median [IQR]	0 [0, 1]	0 [0, 0]	0.791
Delirium, *n (%)*	17 (41.5)	85 (68.6)	0.002
Prevalent, *n (%)*	12 (29.3)	40 (32.3)	0.823
Incident, *n (%)*	5 (12.2)	45 (36.3)	0.006
Hypotension, *n (%)*	11 (26.9)	44 (35.5)	0.390
Tachypnea, *n (%)*	7 (17.1)	44 (35.5)	0.041
Altered mental status, *n (%)*	19 (46.4)	66 (53.2)	0.528
White Blood Cells (×10^3/ μl), mean ± *SD*	17.7 ± 10.5	15.4 ± 9.2	0.222
Hemoglobin (g/dl), mean ± *SD*	12.1 ± 2.1	11.1 ± 2.5	0.015
Platelets Count (× 10^3/μl), mean ± *SD*	256.1 ± 97.1	242.0 ± 114.6	0.443
Creatinine (mg/dl), mean ± *SD*	1.51 ± 0.82	1.80 ± 1.22	0.088
Bilirubin mg/dl), mean ± *SD*	0.8 ± 0.6	0.9 ± 0.8	0.272
Albumin (g/dl), mean ± *SD*	2.8 ± 0.6	2.4 ± 0.8	0.004
pH, mean ± *SD*	7.43 ± 0.08	7.43 ± 0.09	0.972
C Reactive Protein (mg/dl), mean ± *SD*	14.7 ± 8.6	14.7 ± 9.9	0.980
Procalcitonin (ng/ml), mean ± *SD*	25.0 ± 37.0	22.0 ± 33.0	0.673

*Note: SPMSQ* Short Portable Mental Status Questionnaire (number of errors), *BADL* Basic Activities Of Daily Living, *IADL* Instrumental Activities of Daily Living; Hypotension = systolic blood pressure < 100 mmHg; tachypnea = Respiratory Rate ≥ 22/min; altered mental status = Glasgow Coma Scale ≤14

**Fig. 2:**
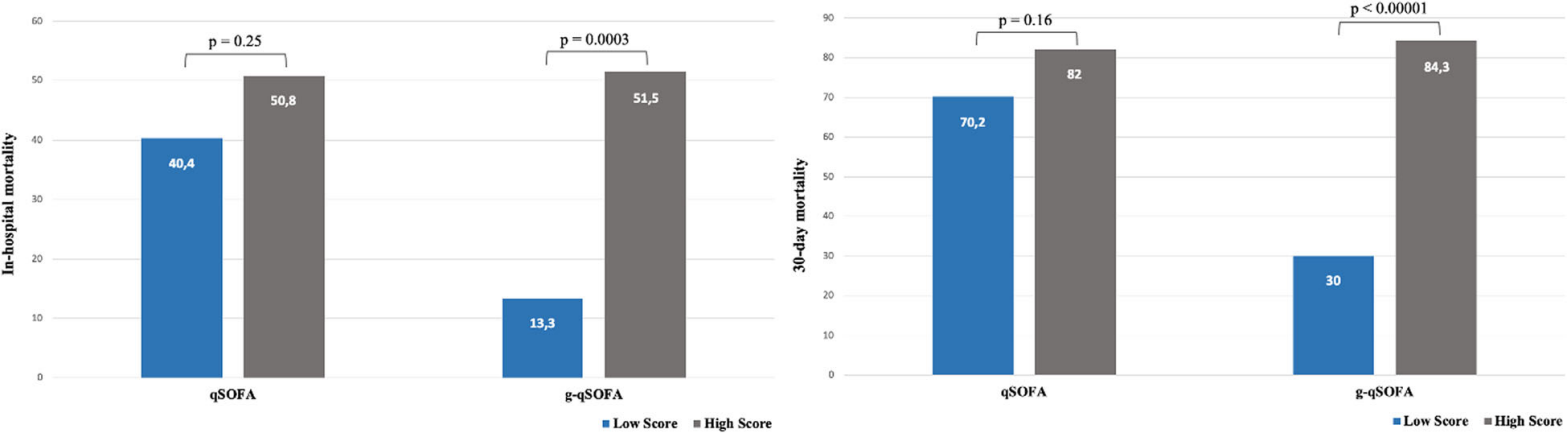
Traditional and geriatric-quickSOFA in relation to in-hospital and 30-day mortality rate

The traditional qSOFA was not significantly associated with 30-day mortality, whereas the new geriatric-quickSOFA was strongly and significantly associated with both 30-day and in-hospital mortality (Fig. 2). Performance indicators of the two score are presented in Table 2. Based on sensibility, accuracy, OR and Youden index results for 30-day mortality, the best cut-off was defined at 1.

**Table 2:** Performance indicators of the traditional qSOFA and geriatric qSOFA

	qSOFA	Geriatric qSOFA
**Sensitivity**	73.2%	92.6%
**Specificity**	40.7%	50.0%
**OR**	1.87	12.5
**Accurancy**	48.8%	81.7%
**PPV**	29.1%	84.3%
**NPV**	81.9%	70.0%
**Youden index**	0.14	0.43

*Note: OR* Odds Ratio, *PPV* Positive Predictive Value, *NPV* Negative Predictive Value

Geriatric-qSOFA score ≥ 1 was significatively related to in-hospital (13.3% vs 51.5%, *p* = 0.0003) and 30-day mortality (30.0% vs 84.3%, *p* < 0.00001) (Fig. 2).

These results were confirmed in multivariable logistic regression analysis, adjusting for age, gender, bedridden status, functional status, and serum hemoglobin and albumin levels (Table 3). Patients with a high geriatric-qSOFA at admission were 15 times more likely to die from sepsis at 30-day, as compared to patients with low geriatric-qSOFA. Furthermore each items of geriatric qSOFA was independently tested in single multivariable analyses adjusted for the same potential confounders: the geriatric qSOFA score had stronger predictive value and better perfomance than each single item in predicting 30-day mortality. In particular, delirium alone was also significantly correlated with the outcome (OR 5.16; 95%CI 2.07–12.9), but the strength of the association was significantly lower as compared to the geriatric-qSOFA. Moreover, geriatric qSOFA showed a higher sensitivity (92.6% versus 57.5%), positive predictive value (84.3% versus 41.5%), accurancy (81.7% versus 68.0%) and Youden index (0.43 versus 0.29) than delirium as single item.

**Table 3:** Multivariate logistic regression analysis for the probability of 30-day mortality

	Model 1 OR (I.C. 95%)	Model 2 OR (I.C. 95%)	Model 3 OR (I.C. 95%)	Model 4 OR (I.C. 95%)	Model 5 OR (I.C. 95%)
g-qSOFA (cut-off ≥1)	11.96 (4.79–29.84)	12.64 (4.89–32.70)	13.23 (4.95–34.77)	11.45 (4.27–30.72)	15.63 (3.88–62.99)
Age	–	0.98 (4.48–2.54)	0.99 (0.37–2.61)	0.98 (0.36–2.62)	0.99 (0.25–3.99)
Male sex	–	1.73 (0.67–4.48)	1.71 (0.65–4.53)	1.70 (0.63–4.56)	2.74 (0.68–11.05)
BADL^a^	–	–	1.03 (0.39–2.74)	1.05 (0.39–2.83)	1.25 (0.31–5.10)
IADL^b^	–	–	1.01 (0.38–2.69)	0.99 (0.37–2.65)	0.75 (0.19–3.02)
Bedridden	–	–	–	2.05 (0.76–5.49)	3.06 (0.76–12.34)
Hemoglobin	–	–	–	**–**	0.89 (0.22–3.61)
Albumin	–	–	–	–	0.19 (0.05–0.76)

^a^n. BADL preserved ^b^n. IADL preserved. Model 1 (crude); Model 2 (+ age and gender); Model 3 (+ BADL and IADL); Model 4 (+ bedridden); Model 5 (+ hemoglobin and albumin)

Results were similar for in-hospital mortality.

## Discussion

In this study of older patients hospitalized with sepsis, original qSOFA was not associated with the risk of death, whereas a modified version, including presence of delirium as indicator of impaired mental status, was significantly associated with both in-hospital and 30-day mortality risk, supporting the hypothesis that qSOFA might not be an adequate prognostic tool in the geriatric population. In multivariable logistic regression analysis, adjusted for potential confounders, patients with a modified qSOFA score ≥ 1 had an almost fifteen-fold risk of death compared to patients with a score of zero.

Our findings extend the results of previous studies providing new insight into the prognostic value of qSOFA in the geriatric population. The ability of qSOFA to predict the clinical course of older patients with sepsis has been already challenged in previous studies [13–17]. Most available studies were performed in ICU with discordant results. While in a study performed on 92 geriatric patients with sepsis admitted to ICU, a significant difference was found in 28-day mortality based on qSOFA value [13], others two recent studies have suggested a low discriminative performance of qSOFA on 30-day (AUC 0.640) [14] and in-hospital mortality (AUC 0.596) [15]. Also in studies conducted in non-ICU wards the prognostic value of qSOFA was not consistent. In a prospective study performed on a sample of 272 patients admitted to Geriatric Ward, qSOFA did not predict in-hospital death and was related only with 3-month mortality [17]. Furthermore, in a recent study on a hospitalized population with median age of 75 years old, qSOFA was not an independent predictor of mortality [19]. On the contrary, in adults qSOFA shows a high sensitivity (87.7%), negative predictive value (96.6%) and accuracy (0.83) [18].

We have therefore developed an alternative qSOFA, respecting the principles under which traditional qSOFA was created (simple and based on clinical parameters without any laboratory tests), but more specifically tailored on older patient characteristics. In particular, we replaced the GCS item with presence of delirium as indicator of mental status impairment, as we hypothesised that delirium would be a more specific marker for the presence of underlying acute disease, as sepsis, and because it is correlated with a very high risk of death in older people [22–25]. Indeed, in addition to advanced age and frequent pre-existing cognitive impairment, presence of sepsis is a well-known trigger for delirium [25–27]. One additional explanation for the better performance of delirium as compared to GCS, initially created to evaluate the severity of impairment of consciousness in neuro-surgical patients with cerebral trauma [28], is that GCS might not be suitable for discriminating between acute and chronic cognitive disfunction including, but not limited to, pre-existing cognitive decline or behavioural and psychological symptoms of dementia. In other words, in acute ill geriatric patients with persistent and pre-existing cognitive dysfunction, GCS assessment might result in a false *positive score,* not being able to distinguish between acute and chronic dysfunction, with a possible consequent inappropriate hospitalization [29].

Based on statistical analysis, the better cut-off of geriatric-qSOFA score was defined at 1: a geriatric-qSOFA ≥1 at hospital admission was associated with an increased risk of in-hospital and 30-day mortality about 8 and 15 times higher as compared to patients with lower score. These estimates were independent of important potential confounders including demographic characteristics, functional status, hemoglobin and albumin levels, or well-known risk factors for mortality in older people. These findings further support the potential utility of geriatric-qSOFA as prognostic tool in geriatric patients with sepsis and multimorbidity.

In interpreting these findings some limitations should be considered. The retrospective observational design of the study might have introduced some degree of classification bias regarding qSOFA and delirium assessment. The small sample size reduced the statistical power of our analyses increasing the likelihood of type II error. Farther, more than 50% of the enrolled population was bedridden before hospital admission and this finding might have disproportionally increased mortality rate, independent of the severity of sepsis, reducing the external validity of our findings. Finally, we developed a modified version of the qSOFA but we were not able to validate it using either an internal or an external independent cohort.

## Conclusion

In conclusion, our study suggests a new prognostic score, specifically tailored for the geriatric population, and designed to predict early mortality from sepsis in this group of patients.

These preliminary data need further investigations and prospective validation of its clinical use as prognostic score using independent cohorts of older population with sepsis.

## Data Availability

The datasets of the current study are available from the corresponding author on reasonable request.

## Abbreviations

GCS: Glasgow Coma Scale
qSOFA: QuickSOFA
g-qSOFA: Geriatric-quickSOFA
ICU: Intensive Care Unit
SPMSQ: Short Portable Mental Status Questionnaire
CCI: Charlson Comorbidity Index
SBP: Systolic Blood Pressure
RR: Respiratory Rate
OR: Odds Ratio
CI: Confidence Interval
BADL: Basic Activities Of Daily Living
IADL: Instrumental Activities of Daily Living
DSM-5th: Diagnostic and Statistical Manual of Mental Disorders 5th Edition criteria
